# Alterations in leukocyte transcriptional control pathway activity associated with major depressive disorder and antidepressant treatment

**DOI:** 10.1038/tp.2016.79

**Published:** 2016-05-24

**Authors:** S H Mellon, O M Wolkowitz, M D Schonemann, E S Epel, R Rosser, H B Burke, L Mahan, V I Reus, D Stamatiou, C -C Liew, S W Cole

**Affiliations:** 1 Department of Obstetrics, Gynecology and Reproductive Sciences, University of California, San Francisco, San Francisco, CA, USA; 2 Department of Psychiatry, University of California, San Francisco, San Franciso, CA, USA; 3 GeneNews Ltd., Richmond Hill, ON, Canada; 4 Department of Medicine, Hematology-Oncology, University of California, School of Medicine, Los Angeles, Los Angeles, CA, USA

## Abstract

Major depressive disorder (MDD) is associated with a significantly elevated risk of developing serious medical illnesses such as cardiovascular disease, immune impairments, infection, dementia and premature death. Previous work has demonstrated immune dysregulation in subjects with MDD. Using genome-wide transcriptional profiling and promoter-based bioinformatic strategies, we assessed leukocyte transcription factor (TF) activity in leukocytes from 20 unmedicated MDD subjects versus 20 age-, sex- and ethnicity-matched healthy controls, before initiation of antidepressant therapy, and in 17 of the MDD subjects after 8 weeks of sertraline treatment. In leukocytes from unmedicated MDD subjects, bioinformatic analysis of transcription control pathway activity indicated an increased transcriptional activity of cAMP response element-binding/activating TF (CREB/ATF) and increased activity of TFs associated with cellular responses to oxidative stress (nuclear factor erythroid-derived 2-like 2, NFE2l2 or NRF2). Eight weeks of antidepressant therapy was associated with significant reductions in Hamilton Depression Rating Scale scores and reduced activity of NRF2, but not in CREB/ATF activity. Several other transcriptional regulation pathways, including the glucocorticoid receptor (GR), nuclear factor kappa-B cells (NF-κB), early growth response proteins 1–4 (EGR1–4) and interferon-responsive TFs, showed either no significant differences as a function of disease or treatment, or activities that were opposite to those previously hypothesized to be involved in the etiology of MDD or effective treatment. Our results suggest that CREB/ATF and NRF2 signaling may contribute to MDD by activating immune cell transcriptome dynamics that ultimately influence central nervous system (CNS) motivational and affective processes via circulating mediators.

## Introduction

Major depressive disorder (MDD) is a pressing public health problem, with up to 16% of the US population experiencing at least one episode in their lives.^1, 2^ It is the leading cause of disability in North America and is projected to become the second leading cause of disability worldwide by 2020.^3, 4, 5, 6, 7^ Identifying circulating biomarkers that parallel disease progression and effective treatment might help illuminate the pathophysiology of MDD and identify distinct subtypes amenable to specific therapies.^8, 9, 10, 11, 12, 13^ Several studies have investigated whether specific profiles of gene expression in circulating leukocytes might provide peripheral diagnostic biomarkers of MDD that could be ascertained easily.^14, 15, 16, 17, 18, 19, 20^ Despite considerable evidence that vulnerability to MDD is partially heritable, to date, leukocyte transcriptome analysis has not shown replicable differences as a function of MDD or its treatment.^14, 15, 16, 17, 18, 19, 21, 22, 23, 24^ However, several studies have linked MDD to activation of upstream gene regulatory pathways that modulate broad patterns of gene expression in immune cells. These pathways include the cAMP response element-binding/activating transcription factor (CREB/ATF) family of transcription factors (TFs),^18, 22^ the glucocorticoid receptor (GR)^25, 26, 27, 28, 29, 30^ and early growth response (EGR) family TFs.^31, 32, 33, 34^ Other studies have linked MDD to altered activity of circulating mediators that regulate TF activity, including glucocorticoids,^35, 36, 37, 38^ pro-inflammatory cytokines, such as interleukin (IL)-1β, IL-6 and tumor necrosis factor-α,^36, 37, 39, 40^ Type I interferons^37, 41, 42, 43^ and oxidative stress.^40, 44, 45, 46^

For both biological and statistical reasons, variations in the activity of signal transduction pathways, rather than small variations in expression of individual downstream gene targets, might show more reliable associations with MDD. We hypothesize that even if correlations between depression and transcriptionally activated pathways exist, it may be difficult to identify significant changes in the expression of individual genes activated through those pathways because of the additional effects of individual differences in genetic background, early developmental influences (for example, epigenetic imprinting) and current physiologic conditions (for example, individual variation in the activity of other hormones or TFs that jointly influence transcription). Statistically, reliability in measurements will increase when levels of expression of individual genes are aggregated into a single measure of common signaling pathway activity. The activity of transcriptional control pathways may also help clarify the biological mechanisms by which gene expression in immune cells correlates with MDD. Regulatory pathway analysis, rather than individual gene analysis, may thus be a more reliable biomarker of MDD and its sequelae.

In the present study, we applied a promoter-based bioinformatic strategy to assess transcriptional control pathway activity via its functional impact on genome-wide transcriptional profiles, to test several emerging theories regarding immune system and oxidative stress involvement in MDD pathophysiology and its remediation by antidepressant treatment. In this bioinformatics approach, differentially expressed genes are first identified, and the promoter DNA sequence of each differentially expressed gene is then scanned to identify the prevalence of TF-binding motifs (TFBMs) that are hypothesized to be activated in association with the condition under study (for example, MDD-associated activation of CREB/ATF TFs, which mediate signaling by many neurotransmitter systems including catecholamines from the sympathetic nervous system). The identification of such 'promoter TFBM enrichment' within a set of differentially expressed genes has been shown in several previous validation studies to provide a generally accurate indication of whether a given TF contributes to the observed difference in gene expression profiles.^47, 48, 49^ The conceptual logic of the analysis is as follows: if a TF is activated, it will only modulate the expression of those genes that bear the specific TFBM to which that TF binds. Thus, the subset of upregulated genes will show a relative enrichment of promoter TFBMs for the activated TF (for example, CREB) relative to that observed in a control set of genes (for example, the basal prevalence across all genes in a genome, or TFBM prevalence in a parallel derived set of downregulated genes, which better controls for the fact that about half of the genome is not expressible in any given cell type because of intrinsic variations in the transcriptional basis for cell development and differentiation). In the present study, we conducted such promoter TFBM enrichment analyses to test a series of *a priori* theories regarding the transcription control pathways that may be activated in the context of MDD, including the CREB/ATF family,^18, 22^ the GR,^25, 26, 27, 28, 29, 30, 50, 51, 52^ EGR family TFs,^31, 32, 33, 34^ the nuclear factor kappa-B cell (NF-κB)/Rel family of TFs that mediate signaling by many pro-inflammatory cytokines,^36, 37, 39, 40^ interferon regulatory factor (IRF) family TFs that mediate signaling by Type I interferons^37, 41, 42, 43, 53^ and the nuclear factor erythroid-derived 2-like 2 (NRF2) TF that mediates transcriptional responses to oxidative stress.^40, 44, 45, 46^

## Materials and methods

### Subjects

We recruited 20 subjects with MDD, diagnosed with the Structured Clinical Interview for DSM-IV-TR (SCID),^54^ and 20 healthy controls matched by sex, ethnicity and age (±3 years; Table 1). These subjects are from the same sample as described previously.^55, 56^ Subjects gave informed consent to participate in this study, which was approved by the University of California, San Francisco Committee on Human Research. The subjects were recruited by fliers, bulletin board notices, Craigslist postings (http://sfbay.craigslist.org), newspaper ads and through clinical referrals. Subjects were seen in an outpatient setting, and were paid for their participation. SCID diagnostic interviews were conducted by an experienced clinical psychologist and were clinically verified by a separate psychiatric interview with a board-certified psychiatrist. Depressed subjects with psychosis, bipolar histories and post-traumatic stress disorder were excluded from participation in the study; other comorbid anxiety disorders were allowed when the depressive diagnosis was considered to be the primary diagnosis. MDD subjects were required to have a rating of 17 or greater on the 17-item Hamilton Depression Rating Scale (HDRS).^57^ Healthy control subjects were also screened with the SCID, and were required to have no present or past history of any DSM-IV Axis I diagnosis. Potential subjects were also excluded if they met SCID criteria for alcohol or substance abuse within 6 months of entering the study. Subjects in both groups were medically healthy (assessed by physical examination, review of systems and screening laboratory tests), had no acute illnesses or infections and had not had any vaccinations within 6 weeks of entering the study. Depressed and control subjects were free of any psychotropic medications, including antidepressants, antipsychotics and mood stabilizers, hormone supplements, steroid-containing birth control or other medications (for example, statins) or vitamin supplements above the US Recommended Daily Allowances (for example, vitamin C, 90 mg per day) for a minimum of 6 weeks before entry into the study; use of short-acting sedative hypnotics was allowed as needed, up to a maximum of three times per week, but none within 1 week before testing.

### Procedure

Subjects were admitted as outpatients to the University of California, San Francisco Clinical and Translational Science Institute’s Clinical Research Center at 0800 hours, having fasted (except water) since 2200 hours the night before. Subjects were required to test negative on a urine toxicology screen (measuring the presence of abused drugs) and, in women of child-bearing capacity, a urine pregnancy test. While subjects sat or reclined in a resting position, blood samples were obtained for leukocyte RNA preparation.

### Treatment

Following the baseline evaluation and blood draw, depressed subjects were treated for 8 weeks, in an open-label manner, with sertraline, dosed according to the clinicians’ judgment, based on efficacy and tolerability, beginning with 50 mg per day, increasing to a maximum of 200 mg per day. In two cases, the beginning dose was lowered to 25 mg per day because of transient side effects. Medication compliance was monitored by pill counts and by plasma levels at weeks 4 and 8 of treatment. The mean plasma concentration of sertraline+*N*-desmethylsertraline at week 4 was 46±23 ng ml^−1^; range: 10–97 ng ml^−1^ and at week 8 was 67±37 ng ml^−1^; range: 10–146 ng ml^−1^. All individuals had plasma concentrations within the range of published steady-state concentrations for sertraline at therapeutic doses,^59^ indicating good compliance with medication treatment. One depressed subject prematurely dropped out of the study because of clinical worsening while on sertraline, and two were excluded because of developing exclusionary criteria unrelated to sertraline (medical illness) during the 8-week follow-up period, leaving 17 treated MDD subjects.

### Gene expression profiling and transcription control bioinformatics

Genome-wide transcriptional profiling was carried out on peripheral blood mononuclear cells (PBMCs) obtained from 20 individuals meeting diagnostic criteria for MDD (HDRS>17) and 20 age- sex- and ethnicity-matched non-depressed controls. All participants were unmedicated at baseline, and 17 MDD subjects subsequently initiated antidepressant treatment and were assessed again for changes in PBMC gene expression profile after 8 weeks of treatment. Blood was collected into vacutainer tubes containing EDTA. RNA was extracted from 10 ml whole blood, after red cell lysis in a hypotonic buffer (150 mm ammonium chloride, 10 mm potassium bicarbonate and 1 mm EDTA) and was incubated on ice for 15 min. Samples were centrifuged at 500 *g* for 10 min at 4 ^o^C, the supernatant was discarded, 1 ml of lysis buffer was added to the pellet and the leukocytes were resuspended by vortexing. The leukocyte suspension was centrifuged, the supernatant was removed, the leukocyte pellet was resuspended in TRIzol Reagent (Invitrogen, Grand Island, NY, USA), extracted with chloroform and RNA was precipitated with isopropanol at −20 ^o^C, followed by centrifugation. The RNA pellet was resuspended in 10 mm Tris-Cl pH 7.5/1 mm EDTA; total RNA was tested for quantity (Nanodrop ND1000, Thermo Scientific, Wilmington, DE, USA), integrity and purity (Agilent Bioanalyzer, Santa Clara, CA, USA). All samples met quality criteria: RIN⩾7.0 ('RIN' is RNA integrity number, a software tool designed to estimate RNA integrity; Agilent Technologies, Santa Clara, CA, USA); 28 S:18 S rRNA ratio⩾1.0. Subsequently, 5 μg of total RNA per sample were used for genome-wide transcriptional profiling using Affymetrix U133 Plus 2 high-density oligonucleotide arrays, following the manufacturer’s protocol (Affymetrix, Santa Clara, CA, USA). Low-level gene expression data were normalized by robust multi-array averaging^60, 61^ and were log_2_-transformed for analysis. Differentially expressed genes were identified by >15% difference in average expression level across groups (that is, a low-stringency screening as recommended for the development of optimally replicable gene lists^62, 63, 64, 65, 66, 67^). Functional characteristics of differentially expressed genes were identified by enrichment analysis of Gene Ontology annotations and Entrez-Gene/RefSeq annotations.^68^ To test the hypothesis that differential gene expression was mediated in part by altered activity of specific TFs, TELiS bioinformatics analysis^47, 62^ analyzed the promoters of differentially expressed genes for over-representation of TFBMs targeted by CREB/ATF factors (TRANSFAC V$CREB_01 position-specific weight matrix)^69^ the GR (V$GR_Q6), NF-κB/Rel factors (V$CREL_01), EGR1–4 (V$EGR1_01, V$EGR2_01, V$EGR3_01, V$NGFIC_01), interferon-responsive TFs (V$ISRE_01) and the oxidative-stress-responsive TF NRF2 (V$NRF2_01). TFBM prevalence was ascertained within the promoters of genes selectively upregulated in association with MDD, and separately within the promoters of genes selectively downregulated in association with MDD (that is, relatively upregulated in controls), and the magnitude of differential TF activity was estimated by the ratio of TFBM prevalence in promoters from upregulated genes versus downregulated genes. This approach ensures that all genes analyzed are potentially expressible within the PBMC pool (that is, are expressed at differentially detectable levels in either MDD or controls), and thereby eliminates potential biases that may stem from using the genome-wide prevalence of promoter TFBMs as a reference point (given that approximately half of the human genome is not materially expressible in PBMC at all because of intrinsic transcriptome biases associated with cell development and differentiation). TFBM ratios were calculated in analyses using nine alternative TFBM scan parameters involving varying signal detection stringency (mat_sim=0.80, 0.90, 0.95)^70^ and promoter length (−300, −600, −1000 to +200 bp relative to the RefSeq-annotated gene transcription start site),^71^ and log-transformed ratios were averaged to estimate TFBM prevalence ratios robust to methodological variations. Average relative prevalence ratios significantly exceeding the null hypothesis of 1.0 indicate greater TFBM prevalence among genes upregulated in association with MDD (that is, relative TF activation in MDD), and average TFBM ratios significantly less than the null hypothesis value of 1.0 indicate lower TFBM prevalence among genes upregulated in association with MDD (that is, relative TF de-activation in MDD, or equivalently, relative TF activation in controls). These bioinformatics indications have been found to provide accurate inferences of TF activity as assessed by direct assays of TF binding (for example, electrophoretic mobility shift assays or nuclear protein enzyme-linked immunosorbent assays) and experimental manipulation of TF activity.^47, 48, 49, 72^ To assess the generality of the present findings, we also repeated TF bioinformatics analyses on data from two archival studies that compared peripheral blood cell RNA samples from MDD patients versus controls (GSE38206) and cancer patients with low versus high levels of depressive symptoms (GSE36957).

### Quantitative PCR analysis of subject RNA

Total RNA was isolated from human samples using Qiagen RNeasy (Qiagen, Valencia, CA, USA) following the recommended protocol supplied with the kit. Lysis buffer was added to solubilize the sample and added to the nucleic acid-binding columns supplied with the kit. RNA bound to the column was washed and eluted, and the concentration of the RNA was measured with a Nanodrop Spectrophotometer (Thermo Scientific, Wilmington, DE, USA).

Invitrogen Superscript III (Life Technologies, Grand Island, NY, USA) was used to generate complementary DNA (cDNA), using the manufacturer’s protocols. Samples with yields close to 4–5 μg of total RNA were used to synthesize cDNA. cDNA reactions containing RNA and random primers were annealed at 50 ^o^C and combined with dNTPs, reaction buffer and Superscript III to synthesize cDNA at 42 ^o^C.

Primers used for quantitative PCR (qPCR; Supplementary Table S1) gave single bands of the appropriate size using agarose gel electrophoresis. For qPCR reactions, the cDNA concentration was normalized to 25 ng per reaction. The iQ SYBR Green Supermix (Bio-Rad Laboratories, Hercules, CA, USA) was used in the qPCR reactions and was monitored using MyiQ iCycler qPCR (Bio-Rad Laboratories). The quality of the PCR reactions was monitored for their Ct values to ensure that data obtained occurred before the reagents were exhausted. To confirm the quality of the data and to ensure that single DNA fragments were generated, PCR amplification products were analyzed using agarose gel electrophoresis at the end of the runs. qPCR reactions were performed in duplicate. The Ct value of the reference gene (*GAPDH*) was monitored as an internal control. Gene expression differences were estimated using the 'Comparative Ct method' of relative quantification, normalizing the C_t_ values relative to the reference gene. This was performed by calculating ΔCt_sample_=Ct_targetgene_−Ct_referencegene_. The relative fold change was represented as 2^-ΔΔCt,^ where ΔΔCt=mean ΔCt_samples_−mean ΔCt_controlsamples_. The amplification efficiencies (close to 100%) of the genes of interest and the housekeeping genes were similar (within 5–10%).

## Results

### Gene expression in MDD versus control

There were no significant differences in the mean age of the depressed versus control subjects, their sex distribution, ethnicity or body mass index (Table 1). The groups also did not differ in current and past alcohol and nicotine consumption, marital status, highest educational level attained, although both self-rated socioeconomic status^58^ and the mean household income were significantly lower in the depressed subjects (*P*<0.04 socioeconomic status; *P*<0.01 household income). Average activity/exercise levels per month, as measured by the Yale Physical Activity Survey,^73^ were not different between groups. The mean 17-item HDRS^57^ rating in the depressed subjects at baseline was 18.71±3.22 and after 8 weeks of antidepressant treatment was 10.24±6.32, *P*<0.001, and the mean chronicity of depression (that is, lifetime years of depression) was 14.02±11.75 years (range 0.77–35 years), corresponding to a mean ratio of lifetime depression to chronological age of 0.36±0.27 (range 0.02–0.88).

In comparisons of depressed individuals with controls, microarray gene expression analysis identified 346 distinct transcripts showing >15% difference in the average expression level across groups (Supplementary Table S2). The 157 transcripts relatively overexpressed in leukocytes from subjects with MDD included multiple genes involved in Type I interferon responses (*IFI44, IFI44L, IFIT1, IFI6, MX1, OAS3, OASL* and *RNASE2*), antimicrobial responses/tissue remodeling (*APOBEC3B, DEFA1, FCER1G, FCGR2B/CD32, MMP8, NOD2* and *PI3*), cytokine and chemokine signaling (*CCL3, CCR3, IL-5RA* and *IL-18*), immunoglobulin production (*IGJ* and *IGH@*), cellular activation and proliferation (*HLA-DR, HLA-DQ, IGF1R, PTEN* and *EGR3*), skewing of macrophage responses toward M2 profiles (*ARG1*) and cellular responses to oxidative stress (*GSTM4*). Prominent among the 189 downregulated transcripts in subjects with MDD were genes involved in cAMP/PKA signaling (*PRKACB, PRKAR2B* and *PDE3B*), genes encoding antibody immunoglobulin heavy chains (*IGHD* and *IGHM*) and antibody receptors (*FCRL1, FCRL3, FCRL5/CD307* and *FCRLA*), the T-cell receptor alpha gene (*TRA@*), chemokine receptors (*CCR7* and *CXCR6*), multiple leukocyte cell surface antigens (*CD6, CD7, CD22, CD79A* and *LY9/CD229*) and *EGR1*. The prominent differential expression of leukocyte cell type-specific transcripts suggested potential alterations in subset distribution within the leukocyte pool in MDD versus control subjects. However, we found no significant difference in the leukocyte pool level of mRNA for any canonical leukocyte subset marker (*CD14, CD19, CD16/FCGR3A CD56/NCAM1, CD3, CD4* and *CD8*; all average differences <15%, all *P*>0.10).

### Transcription control pathways in MDD versus control

Promoter-based bioinformatics analyses (Table 2) showed significant over-representation of response elements for CREB/ATF factors and the master antioxidant TF NRF2 in the promoters of genes upregulated in association with MDD. Results also indicated nominally significant and unanticipated under-representations of NF-κB and EGR2 response elements in promoters of genes upregulated in association with MDD, although these latter two findings would not attain significance under Bonferroni correction for multiple testing across the nine TFBMs analyzed. Primary analyses showed no significant indication of GR, EGR1, EGR3 or EGR4 activation in MDD versus control subjects. Ancillary analyses adjusting gene expression data for age, sex, body mass index and the fractional distribution of lymphocytes, monocytes and eosinophils in the assayed leukocyte pool (Table 2) continued to indicate over-representation of CREB/ATF and NRF2 response elements in promoters of genes upregulated in association with MDD. Several additional TFBM signals also reached significance in the adjusted analyses, including under-representation of EGR2, EGR3 and interferon-simulated response element TFBMs, and over-representation of EGR1 motifs (although the latter two results would not reach significance under Bonferroni correction).

### Gene expression changes with MDD treatment

After 8 weeks of antidepressant therapy, average HDRS depression scores were reduced significantly from baseline levels in MDD subjects (baseline: 18.71±3.22; week 8: 10.24± 6.32, *P*<0.001). Transcriptional profiling of leukocytes collected after 8-week treatment identified 183 transcripts showing >15% upregulation relative to baseline and 101 showing >15% downregulation (Supplementary Table S3). Prominent among upregulated transcripts were gene products involved in Type I interferon response, including several that were upregulated in the MDD versus control baseline comparison (marked with *); these were IFI27, IFI44*, IFI44L*, IFI6*, IFIT1, IRF7, ISG15, OAS1, OAS2, OAS3* and OASL*. Multiple transcripts associated with the myeloid lineage of immune cells were also upregulated, including *CD36, LY6E* and the macrophage-associated TF *MAFB*. However, transcripts associated with macrophage activation also numbered among the downregulated transcripts, including several that were upregulated in leukocytes from MDD versus control subjects at baseline (for example, APOBEC3B*, ARG1*, IL-1R1, MMP8, PI3* and XIST*). Downregulated transcripts also included the oxidative stress response gene *SOD2*.

### Transcription control pathways in MDD after treatment

Promoters of genes that were downregulated following antidepressant treatment (Table 2) showed significant over-representation of NRF2 TFBMs, whereas those that were upregulated following antidepressant treatment showed over-representation of interferon-related TFBMs. Analyses also linked treatment-related changes in gene expression to TFBM prevalence patterns indicative of increased NF-κB activity and decreased EGR2 and EGR3 activity (although these latter three counter-hypothetical findings would not reach statistical significance under Bonferroni correction). Primary analyses showed no significant indications of CREB, GR, EGR1 or EGR4 involvement in treatment-related changes in gene expression. Ancillary analyses adjusting for age, sex and leukocyte subset distribution in the leukocyte pool showed similar results, with the notable difference that CREB/ATF, EGR1, EGR2 and EGR3 TFBMs also become significantly over-represented among upregulated promoters (Table 2).

### Confirmatory studies

The microarray data showing differences between RNA abundance in leukocytes from healthy control and MDD subjects were confirmed by direct amplification and quantitation with quantitative reverse transcriptase PCR (RT-PCR) analysis (primers shown in Supplementary Table S1). Among the 11 transcripts re-tested with RT-PCR, nine showed significant differences in expression that matched those indicated by microarray results (Supplementary Table S4).

To assess the generality of the TF bioinformatics findings from this sample, we compared results from contrasting MDD patients versus controls with results from parallel analyses of peripheral blood cell RNA in an archival data set comparing MDD versus controls in another population (GSE38206) and cancer patients with high versus low levels of depressive symptoms (GSE36957). Results (Table 3) showed good concordance for the primary positive results reported above for CREB/ATF and NRF2. Genes upregulated in archival comparison of MDD versus controls also showed significant over-representation of CREB and NRF2 TFBMs, and genes upregulated in cancer patients with high levels of depressive symptoms showed significant over-representation of CREB/ATF TFBMs and marginally significant (*P*=0.059) over-representation of NRF2 TFBMs. Results from these two archival data sets were also consistent among themselves in indicating elevated prevalence of NF-κB and interferon-responsive TFBMs (results not identified in analyses of the present sample). Analysis of the archival MDD versus control comparison also indicated highly significant signals for all of the other pathways analyzed; however, these indications were not corroborated in the cancer/depressive symptoms' sample.

## Discussion

The present analyses utilize a transcriptome-driven bioinformatic strategy to evaluate the activity of several transcriptional control pathways that have been hypothesized to be activated in association with MDD. Results comparing unmedicated MDD subjects versus control subjects support previously proposed roles for CREB and the oxidative stress response factor NRF2. However, only NRF2 showed a significant reduction in activity following 8 weeks of antidepressant therapy. Indices of CREB-mediated gene transcription were not reversed by antidepressant therapy, and continued to trend upward after 8 weeks of sertraline treatment. Thus, the NRF2 transcriptional pathway is distinct from the other candidate transcriptional control pathways evaluated here, as it tracked with both disease (MDD) and effective antidepressant treatment.

These analyses do not support a role for activation of the master pro-inflammatory TF NF-κB, interferon regulatory factors, the GR or the EGR family of TFs in the basic pathogenesis of MDD. Significant associations did emerge for EGR family factors in analyses adjusting for age, sex and leukocyte subset distributions; however, these effects trended in the opposite direction of previous hypotheses (that is, under-representation in MDD-associated and treatment-responsive promoters). Because our sample size is small, and thus has limited statistical power to detect small effects, we cannot conclude that our results definitively refute roles for NF-κB, interferons or the GR in the pathophysiology of depression. Indeed, altered hypothalamic pituitary axis activity may be restricted mainly to the subset of MDD cases associated with trauma.^74^ However, our failure to see positive signals for NFκB and GR activation, when other transcription control pathways such as CREB/ATF and NRF2 show detectable differences in activity, suggests that NF-κB and GR do not constitute the most sensitive or clinically useful TF pathway biomarkers of MDD.

Our results have significant implications for five published hypotheses regarding peripheral immune system function in the genetic basis of MDD. First, it has been proposed^75, 76, 77, 78, 79^ that MDD is associated with reductions in tonic CREB activity, possibly secondary to reduced activity of neurotrophic factors (for example, brain-derived neurotrophic factor) and/or neurotransmitters (for example, adrenergic signaling from catecholamines). Alterations in CREB activity in the central nervous system (CNS) in animal models of depression^77, 78, 80, 81, 82, 83, 84, 85^ and findings from clinical studies linking increased leukocyte CREB activity to successful behavioral or antidepressant therapy^22, 75, 76, 86, 87, 88, 89^ support this view. However, our results find no tonic inhibition of CREB/ATF-mediated gene expression in leukocytes from untreated MDD patients versus control subjects, but rather indicate upregulation of CREB/ATF activity at baseline before treatment. Moreover, comparisons of CREB/ATF-associated gene expression before and after antidepressant treatment are consistent with previous observations that successful MDD treatment is associated with increased CREB phosphorylation in leukocytes.^79, 90, 91^ Thus, although the pathophysiologic role of leukocyte CREB activity remains uncertain, our results are consistent with previous studies, suggesting that increasing leukocyte CREB activity levels may potentially serve as a biomarker of treatment response. Our data are also consistent with studies, suggesting that the mRNAs for ATF3 TFs are increased in MDD compared with healthy controls.^31^

A second hypothesis suggests that dysregulated activity of innate immune response signaling pathways may activate CNS 'sickness behavior' systems that trigger motivational deficits and affective alterations characteristic of MDD.^41, 42, 43^ Administration of Type I interferons induces symptoms of major depression, suggesting that some cases of depression may stem from brain responses to subclinical viral infections or other physiologic dynamics that elicit systemic Type I interferon signaling (for example, reviewed in Raison and Miller^9^ and Loftis *et al.*^92^). Our results are partially consistent with this hypothesis, as they show increased expression of several individual interferon-responsive genes in untreated MDD subjects, including genes involved in the interferon-mediated 'antiviral state'. However, TFBM analyses found no evidence of an aggregate-level elevation in interferon-responsive TF activity in unmedicated MDD patients relative to healthy controls. Moreover, genes that were downregulated in association with antidepressant treatment showed a significant under-representation of interferon-related TFBMs, and individual interferon target transcripts continued to show an upward trend during treatment. This treatment-associated upregulation of interferon signaling activity is consistent with previous observations of increased activation of the interferon response factor 7 TF following citalopram treatment.^93^ These data suggest that if subclinical infectious dynamics or other immunoregulatory disturbances underlie the aberrant interferon signaling associated with some cases of MDD, antidepressants may potentially block the downstream neurobiological consequences of interferon activity, but they are not likely to have an impact on the underlying infectious/physiologic elicitors of interferon activity. This interpretation would be consistent with studies showing that antidepressant prophylaxis can block the emergence of depressive symptoms and sickness behavior following pharmacologic interferon treatment.^41, 42, 43, 94, 95, 96, 97, 98, 99, 100, 101^

Third, the 'macrophage theory of depression' suggests that upregulated monocyte/macrophage production of pro-inflammatory cytokines such as IL-1β, tumor necrosis factor-α and IL-6 activates CNS sickness behavior functions in a manner analogous to that hypothesized for Type I interferons.^102, 103, 104^ This hypothesis would predict that MDD should be linked to TFs involved in monocyte/macrophage activation such as NF-κB and EGR family factors. Although our study replicated previous observations of increased EGR3 mRNA in leukocytes from MDD patients, analyses of NF-κB and EGR TFBM distributions provide no evidence that EGR3, other EGR family members or NF-κB/Rel family members show increased transcriptional activity in MDD. By contrast, we found modest indications of baseline *reductions* in NF-κB- and EGR3-mediated gene expression in leukocytes from MDD patients. These results are not likely to differ from previous findings because of differences in the cell types examined (leukocytes versus isolated monocytes in previous studies) because our adjusted analyses controlled for the relative prevalence of monocytes in the assayed leukocyte pool and because our present study replicated previous indications of increased EGR3 mRNA. Nevertheless, our results suggest reduced functional activity of that pathway. The differing conclusions likely stem from our study’s focus on TF activity, whereas previous studies only focused on EGR3 mRNA abundances. This distinction is consistent with data in other systems showing that TF mRNA abundances do not correlate perfectly with functional TF activity, which can also be substantially regulated by post-translational modifications of TFs.^105^

A fourth hypothesis is that peripheral oxidative stress signals to the CNS to evoke symptoms of depression.^55, 100, 106, 107, 108, 109, 110, 111, 112, 113, 114, 115, 116, 117, 118, 119, 120, 121, 122^ Our results are highly consistent with that hypothesis. We found baseline indications of increased activity for the oxidative stress response TF NRF2 in MDD, and reduced NRF2 transcriptional activity following antidepressant treatment. The mRNA abundance of SOD2, a redox-sensitive enzyme, also declined following antidepressant treatment. Those effects could not be attributed to differences in body mass index, age or other demographic variables. The implication of NRF2 and SOD2 in altered leukocyte transcription in MDD subjects suggests a potential leukocyte gene transcriptional biomarker in MDD, a potential target for therapeutic intervention, and a potential biomarker of effective treatment. Given that NRF2 emerged as the only TF in our analysis that directly tracked both depression under basal conditions and the effects of antidepressant therapy, identification of the etiology of peripheral reactive oxygen species in MDD and its relationship to other immune-related transcriptional dynamics (for example, type I interferon signaling, NF-κB activity, CREB activity and so on) may provide deeper insights into the biological basis for depression.

A fifth hypothesis that is not supported by our study suggests that chronically high cortisol levels, and consequent alterations in glucocorticoid-responsive transcription contribute to the pathogenesis of MDD.^8, 29, 36, 37, 52, 86, 103, 123, 124, 125, 126, 127, 128, 129, 130^ We found no significant alterations in GR-mediated gene expression in control versus untreated MDD subjects, either before or after 8 weeks of antidepressant treatment. Because the GR hypothesis primarily concerns glucocorticoid activity in the CNS, our data from circulating leukocytes cannot be taken as a direct contradiction of this hypothesis. However, our results suggest that leukocyte GR activity may not be a useful proxy biomarker of alterations in glucocorticoid response in the CNS.

Our data provide new insights into the potential immunobiological and oxidative processes that might contribute to, or serve as biomarkers of, the neurobiological substrates of MDD. However, our study has several limitations, especially small sample size. Replication with larger samples will be needed to resolve small effects that might remain undetected in the present study. Indeed, such power limitations may explain why some significant TFBM patterns that emerged in analyses of the archival data sets were not confirmed in the present sample (for example, linking MDD to activation of NF-κB and interferon signaling pathways). However, this study’s primary findings regarding CREB/ATF and NRF TFs did prove replicable in the two independent archival data sets. Our target gene mRNA read-out provides a genome-wide functional framework for assessing TF activity. However, we inferred TF activity from promoter sequence analyses; future studies may use more direct assays of TF activity, such as chromatin immunoprecipitation or nuclear protein assays.^49, 72^ Our bioinformatic findings can suggest targets for such analyses, but do not provide direct indications of TF functional activity (for example, DNA binding or transcriptional induction). Our study is also not powered to discover statistically significant associations between individual gene transcripts and MDD versus control status or sertraline treatment. The results in Supplementary Tables S2 and S3 are not tested for statistical significance at the level of specific individual transcript–phenotype associations. Our use of leukocytes as the target cell pool is a strength, in that these cells are readily available, but leukocytes constitute a heterogeneous mix of cells, so that future studies will need to isolate specific leukocyte subtypes to define the specific cell type(s) mediating the observed effects. However, most of the genes that were differentially expressed were from the myeloid lineage, such as monocytes and dendritic cells, which have been implicated in other studies of MDD-associated transcriptional alteration^29, 43^ as well as in studies of socioenvironmental conditions that can precipitate depression.^49, 131, 132, 133, 134^

Our results provide valuable new insights into the pathobiology of MDD by confirming that cells of the immune system show coherent and highly focal transcriptional alterations in association with MDD and effective antidepressant treatment. These results add to a burgeoning literature implicating immune system dynamics in MDD, and they provide information that helps to discriminate among several of the most prominent current theories of MDD. Future studies should address the roles of oxidative stress and CREB/ATF signaling in crosstalk between the peripheral immune system and CNS biology in the context of MDD.

## Figures and Tables

**Table 1:** Characteristics of depressed and control subjects

	*Controls (*n=*20)*	*Depressed (*n=*20)*	P
Age (years)	36.6±11.8	37.0±10.8	ns
Sex (% female)	65%	65%	ns
Ethnicity (% Caucasian, African-American, Asian, other or mixed)	75%, 15%, 5%, 5%	70%, 10%, 15%, 5%	ns
Body mass index	24.7±3.7	24.8±4.3	ns
No tobacco ever (%)	55%	50%	ns
Current tobacco use (%)	20%	35%	ns
Subjective socioeconomic status^a^	6.6±1.0	5.4±1.8	<0.04
Years of education	14.8±2.2	14.7±2.0	ns
Household income ($)	$59 400±$46 500	$28 450±$24 600	<0.02

a Subjective socioeconomic status was measured using a 10-rung ladder version of the MacArthur Scale of Subjective Social Status.^58^

**Table 2:** Promoter transcription factor-binding motif distributions for candidate transcription control pathways

	*MDD versus Control*		*MDD baseline versus antidepressant Tx*	
	*Observed*	*Adjusted*^a^	*Observed*	*Adjusted*^a^
CREB	2.66±0.20, *P*=0.0007^b^	2.40±0.17, *P*=0.0005^b^	1.04±0.10, *P*=0.6872	2.63±0.12, *P*=0.0001^b^
ISRE	1.46±0.39, *P*=0.3091	0.64±0.17, *P*=0.0482	3.14±0.20, *P*=0.0003^b^	4.17±0.36, *P*=0.0098
NRF2	2.11±0.18, *P*=0.0019^b^	1.72±0.09, *P*=0.0003^b^	0.61±0.08, *P*=0.0002^b^	0.48±0.14, *P*=0.0005^b^
NF-κB	0.92±0.03, *P*=0.0480	0.96±0.04, *P*=0.3067	1.43±0.12, *P*=0.0115	0.81±0.06, *P*=0.0078
GR	1.05±0.06, *P*=0.4198	1.01±0.06, *P*=0.9164	0.91±0.10, *P*=0.3437	0.82±0.14, *P*=0.1747
EGR1	1.10±0.10, *P*=0.4216	1.34±0.09, *P*=0.0230	0.83±0.08, *P*=0.0685	2.59±0.21, *P*=0.0042^b^
EGR2	0.42±0.23, *P*=0.0140	0.70±0.08, *P*=0.0017^b^	0.82±0.09, *P*=0.0483	1.67±0.13, *P*=0.0025^b^
EGR3	0.41±0.43, *P*=0.0665	0.58±0.65, *P*=0.0016^b^	0.69±0.13, *P*=0.0139	2.86±0.12, *P*=0.0001^b^
EGR4/NGFIC	0.53±0.36, *P*=0.1113	0.82±0.16, *P*=0.2233	0.89±0.09, *P*=0.2128	2.47±0.25, *P*=0.0101

Abbreviations: CREB, cAMP response element-binding; EGR, early growth response; GR, glucocorticoid receptor; ISRE, interferon-stimulated response element; MDD, major depressive disorder; NF-κB, nuclear factor kappa-B; NRF2, nuclear factor erythroid-derived 2-like 2; PBMC, peripheral blood mononuclear cell; TFBM, transcription factor-binding motif.

Values represent the mean fold difference in promoter TFBM distribution±s.e., and two-tailed *P*-value testing null hypothesis 1.0-fold

.

a Analyses controlling for age, sex and PBMC % lymphocytes, monocytes and eosinophils.

b Statistically significant under Bonferroni correction for simultaneous testing of nine pathways (individual *P*<0.0056).

**Table 3:** Relationship of present findings to analyses of archival data sets

	*MDD versus control*			
	*Observed*	*GSE38206*	*GSE36957*	*Relationship*^a^
CREB	2.66±0.20, *P*=0.0007^b^	1.23±0.03, *P*<0.0001^b^	1.55±0.08, *P*<0.0001^b^	Consistent
ISRE	1.46±0.39, *P*=0.3091	1.81±0.17, *P*=0.0003^b^	1.46±0.23, *P*=0.0030^b^	Additional
NRF2	2.11±0.18, *P*=0.0019^b^	1.42±0.05, *P*<0.0001^b^	1.06±0.04, *P*=0.0593	Partially consistent
NF-κB	0.92±0.03, *P*=0.0480	1.06±0.03, *P*=0.0016^b^	1.29±0.09, *P*<0.0001^b^	Additional
GR	1.05±0.06, *P*=0.4198	0.84±0.07, *P*=0.0033^b^	0.83±0.10, *P*=0.0657	Null
EGR1	1.10±0.10, *P*=0.4216	1.59±0.13, *P*=0.0010^b^	1.07±0.03, *P*=0.2893	Null
EGR2	0.42±0.23, *P*=0.0140	1.96±0.24, *P*=0.0004^b^	0.98±0.05, *P*=0.6717	Null
EGR3	0.41±0.43, *P*=0.0665	1.75±0.14, *P*=0.0002^b^	1.04±0.09, *P*=0.4854	Null
EGR4/NGFIC	0.53±0.36, *P*=0.1113	1.78±0.18, *P*=0.0008^b^	1.21±0.23, *P*=0.0629	Null

Abbreviations: CREB, cAMP response element-binding; EGR, early growth response; GR, glucocorticoid receptor; ISRE, interferon-stimulated response element; MDD, major depressive disorder; NF-κB, nuclear factor kappa-B; NRF2, nuclear factor erythroid-derived 2-like 2; TFBM, transcription factor-binding motif.

Values represent the mean fold difference in promoter TFBM distribution±s.e., and two-tailed *P*-value testing null hypothesis 1.0-fold.

a Consistent, archival studies are consistent and are in concordance with results of present MDD versus control comparison; Additional, archival studies are consistent, but relationship is not detected in present sample; Partially consistent, archival studies are inconsistent, but one is in concordance with present MDD versus Control comparison; Null, archival studies are inconsistent.

b Statistically significant under Bonferroni correction for simultaneous testing of nine pathways (individual *P*<0.0056).
