# Supplementary material for: Alterations in leukocyte transcriptional control pathway activity associated with major depressive disorder and antidepressant treatment
Source: Transl Psychiatry. 2016 May 24;6(5):e821–. doi: 10.1038/tp.2016.79 (PMC5070063; doi:10.1038/tp.2016.79)
Supplement: Supplementary Table 1 [file tp201679x1.docx]

**Supplemental Table S1: Primers used for qPCR analysis of leukocyte RNA**

| GENE | FWD | REV | Size (bp) |
| --- | --- | --- | --- |
| DDX17 | TTGGCCGAACAGCCCGTAGC | GACCACCCTTACCCCCGCCT | 180 |
| DSC2 | CTGTGGGCGCTTCTGCTCAGG | ATGGATTCTTCACCAAGACGGGGC | 238 |
| EGR1 | AGCAGCACCTTCAACCCTCAGG | GAGTGGTTTGGCTGGGGTAACT | 104 |
| FAM118 | TGAAGTACGGCGTCCTCCACAT | GGTGCGGTATAAGTTCTGGAGG | 106 |
| IFI44 | TGGTACATGTGGCTTTGCTC | CCACCGAGATGTCAGAAAGAG | 111 |
| IFI44L | TGCACTGAGGCAGATGCTGCG | TCATTGCGGCACACCAGTACAG | 115 |
| MX1 | GGCTGTTTACCAGACTCCGACA | CACAAAGCCTGGCAGCTCTCTA | 102 |
| PRKAR2B | AACCGATTCACAAGGCGTGCCT | CAGCAGGATGTCTTTGCAAGCC | 122 |
| APOBEC3B | CCTCTATGGTCGGAGCTACACT | GAGGAAGCACATTTCTGCGTGG | 133 |
| MMP8  XIST | CAACCTACTGGACCAAGCACAC  GAAGCACCTGCCAGCAACAGC | TGTAGCTGAGGATGCCTTCTCC  GTCCCCTTGGGACCTCGCTT | 106  126 |
