# Supplementary material for: Alterations in leukocyte transcriptional control pathway activity associated with major depressive disorder and antidepressant treatment
Source: Transl Psychiatry. 2016 May 24;6(5):e821–. doi: 10.1038/tp.2016.79 (PMC5070063; doi:10.1038/tp.2016.79)
Supplement: Supplementary Table 2 [file tp201679x2.docx]

**Supporting Information Table S2: Transcripts differentially expressed in MDD vs Healthy Control Subjects**

| **Probe Set ID** | **Gene Symbol** | **Fold-difference*** | |
| --- | --- | --- | --- |
|  |  |  |  |
| 209480_at | HLA-DQB1 | 3.29 |  |
| 213831_at | HLA-DQA1 | 2.47 |  |
| 205048_s_at | PSPH | 2.18 |  |
| 206632_s_at | APOBEC3B | 2.03 |  |
| 236203_at | HLA-DQA1 | 2.00 |  |
| 212999_x_at | HLA-DQB1 | 1.73 |  |
| 226303_at | PGM5 | 1.71 |  |
| 1558048_x_at | --- | 1.69 |  |
| 217104_at | ST20 | 1.55 |  |
| 224559_at | MALAT1 | 1.51 |  |
| 204351_at | S100P | 1.49 |  |
| 209875_s_at | SPP1 | 1.47 |  |
| 203962_s_at | NEBL | 1.47 |  |
| 224590_at | XIST | 1.42 |  |
| 217427_s_at | HIRA | 1.42 |  |
| 220066_at | NOD2 | 1.41 |  |
| 211517_s_at | IL5RA | 1.40 |  |
| 1557051_s_at | --- | 1.40 |  |
| 228642_at | --- | 1.40 |  |
| 1557050_at | --- | 1.40 |  |
| 224489_at | KIAA1267 /// LOC100294337 | 1.40 |  |
| 220421_at | BTNL8 | 1.40 |  |
| 1560263_at | --- | 1.39 |  |
| 228582_x_at | --- | 1.38 |  |
| 204751_x_at | DSC2 | 1.38 |  |
| 227474_at | LOC654433 | 1.38 |  |
| 212509_s_at | MXRA7 | 1.36 |  |
| 204439_at | IFI44L | 1.36 |  |
| 239647_at | CHST13 | 1.35 |  |
| 224490_s_at | KIAA1267 /// LOC100294337 | 1.34 |  |
| 211654_x_at | HLA-DQB1 | 1.34 |  |
| 233217_at | --- | 1.33 |  |
| 224588_at | XIST | 1.31 |  |
| 209135_at | ASPH | 1.31 |  |
| 213478_at | RP1-21O18.1 | 1.30 |  |
| 206111_at | RNASE2 | 1.30 |  |
| 214453_s_at | IFI44 | 1.30 |  |
| 1555116_s_at | SLC11A1 | 1.30 |  |
| 201163_s_at | IGFBP7 | 1.30 |  |
| 224412_s_at | TRPM6 | 1.29 |  |
| 235059_at | RAB12 | 1.29 |  |
| 222646_s_at | ERO1L | 1.29 |  |
| 238900_at | HLA-DRB1 /// HLA-DRB3 /// HLA-DRB4 /// HLA-DRB5 /// LOC100294036 | 1.29 |  |
| 224996_at | ASPH | 1.29 |  |
| 242625_at | RSAD2 | 1.29 |  |
| 226817_at | DSC2 | 1.28 |  |
| 204724_s_at | COL9A3 | 1.28 |  |
| 227671_at | XIST | 1.28 |  |
| 217513_at | C17orf60 | 1.28 |  |
| 1552348_at | PRSS33 | 1.28 |  |
| 212998_x_at | HLA-DQB1 /// LOC100294318 | 1.28 |  |
| 1552349_a_at | PRSS33 | 1.28 |  |
| 203691_at | PI3 | 1.27 |  |
| 214218_s_at | XIST | 1.27 |  |
| 219863_at | HERC5 | 1.27 |  |
| 205844_at | VNN1 | 1.27 |  |
| 212592_at | IGJ | 1.27 |  |
| 203021_at | SLPI | 1.26 |  |
| 217022_s_at | IGH@ /// IGHA1 /// IGHA2 /// LOC100126583 | 1.26 |  |
| 224589_at | XIST | 1.26 |  |
| 218498_s_at | ERO1L | 1.26 |  |
| 41469_at | PI3 | 1.26 |  |
| 230333_at | --- | 1.26 |  |
| 232600_at | --- | 1.26 |  |
| 1552552_s_at | CLEC4C | 1.26 |  |
| 206115_at | EGR3 | 1.25 |  |
| 1554663_a_at | NUMA1 | 1.25 |  |
| 203961_at | NEBL | 1.25 |  |
| 214590_s_at | UBE2D1 | 1.25 |  |
| 209369_at | ANXA3 | 1.25 |  |
| 1556277_a_at | PAPD4 | 1.25 |  |
| 1562777_at | --- | 1.25 |  |
| 206207_at | CLC | 1.25 |  |
| 233425_at | --- | 1.24 |  |
| 228834_at | TOB1 | 1.24 |  |
| 219519_s_at | SIGLEC1 | 1.24 |  |
| 228372_at | C10orf128 | 1.24 |  |
| 213797_at | RSAD2 | 1.24 |  |
| 235764_at | --- | 1.24 |  |
| 215891_s_at | GM2A | 1.24 |  |
| 204750_s_at | DSC2 | 1.24 |  |
| 235761_at | --- | 1.24 |  |
| 1558504_at | --- | 1.24 |  |
| 225008_at | ASPH | 1.24 |  |
| 230048_at | --- | 1.24 |  |
| 204443_at | ARSA | 1.24 |  |
| 207328_at | ALOX15 | 1.23 |  |
| 226702_at | CMPK2 | 1.23 |  |
| 231688_at | MMP8 | 1.23 |  |
| 203153_at | IFIT1 | 1.23 |  |
| 223599_at | TRIM6 | 1.23 |  |
| 229144_at | RP1-21O18.1 | 1.23 |  |
| 205003_at | DOCK4 | 1.23 |  |
| 208253_at | SIGLEC8 | 1.23 |  |
| 225252_at | SRXN1 | 1.22 |  |
| 221728_x_at | XIST | 1.22 |  |
| 241098_at | CLEC7A | 1.22 |  |
| 205041_s_at | ORM1 /// ORM2 | 1.22 |  |
| 205033_s_at | DEFA1 /// DEFA1B /// DEFA3 | 1.22 |  |
| 235818_at | VSTM1 | 1.22 |  |
| 204714_s_at | F5 | 1.21 |  |
| 205513_at | TCN1 | 1.21 |  |
| 203887_s_at | THBD | 1.21 |  |
| 204713_s_at | F5 | 1.21 |  |
| 220384_at | TXNDC3 | 1.21 |  |
| 216627_s_at | B4GALT1 | 1.21 |  |
| 228933_at | NHS | 1.21 |  |
| 204415_at | IFI6 | 1.21 |  |
| 202430_s_at | PLSCR1 | 1.21 |  |
| 234664_at | LOC284701 | 1.21 |  |
| 243824_at | --- | 1.21 |  |
| 210896_s_at | ASPH | 1.20 |  |
| 1558549_s_at | VNN1 | 1.20 |  |
| 242598_at | --- | 1.20 |  |
| 1554899_s_at | FCER1G | 1.20 |  |
| 218400_at | OAS3 | 1.20 |  |
| 239888_at | --- | 1.20 |  |
| 229028_s_at | ARL17 | 1.20 |  |
| 210630_s_at | RAD52 | 1.20 |  |
| 205660_at | OASL | 1.20 |  |
| 1555091_at | PPM1F | 1.20 |  |
| 235816_s_at | RGL4 | 1.20 |  |
| 235408_x_at | ZNF117 | 1.20 |  |
| 235568_at | C19orf59 | 1.20 |  |
| 206295_at | IL18 | 1.20 |  |
| 213506_at | F2RL1 | 1.20 |  |
| 215573_at | CAT | 1.20 |  |
| 227347_x_at | HES4 | 1.19 |  |
| 203780_at | MPZL2 | 1.19 |  |
| 237340_at | SLC26A8 | 1.19 |  |
| 217823_s_at | UBE2J1 | 1.19 |  |
| 210797_s_at | OASL | 1.19 |  |
| 231029_at | F5 | 1.19 |  |
| 236154_at | QKI | 1.19 |  |
| 207329_at | MMP8 | 1.19 |  |
| 218963_s_at | KRT23 | 1.19 |  |
| 213201_s_at | TNNT1 | 1.19 |  |
| 221992_at | NPIPL2 | 1.19 |  |
| 218729_at | LXN | 1.18 |  |
| 1556357_s_at | ERICH1 | 1.18 |  |
| 243352_at | ALPK1 | 1.18 |  |
| 240131_at | --- | 1.18 |  |
| 241843_at | SNORA28 | 1.18 |  |
| 211764_s_at | UBE2D1 | 1.18 |  |
| 205746_s_at | ADAM17 | 1.18 |  |
| 202446_s_at | PLSCR1 | 1.18 |  |
| 236660_at | --- | 1.18 |  |
| 232034_at | LOC203274 | 1.18 |  |
| 225750_at | --- | 1.18 |  |
| 1555144_at | ARL17 | 1.18 |  |
| 242003_at | ERICH1 | 1.18 |  |
| 228425_at | LOC654433 | 1.18 |  |
| 236422_at | --- | 1.18 |  |
| 1562307_at | --- | 1.18 |  |
| 222668_at | KCTD15 | 1.18 |  |
| 241403_at | CLK4 | 1.18 |  |
| 204560_at | FKBP5 | 1.18 |  |
| 205483_s_at | ISG15 | 1.18 |  |
| 1569004_at | --- | 1.18 |  |
| 203627_at | IGF1R | 1.18 |  |
| 226189_at | ITGB8 | 1.17 |  |
| 205386_s_at | MDM2 | 1.17 |  |
| 206177_s_at | ARG1 | 1.17 |  |
| 204149_s_at | GSTM4 | 1.17 |  |
| 236337_at | SYCP2L | 1.17 |  |
| 242622_x_at | PTEN | 1.17 |  |
| 206429_at | F2RL1 | 1.17 |  |
| 235922_at | --- | 1.17 |  |
| 214321_at | NOV | 1.17 |  |
| 207117_at | ZNF117 | 1.17 |  |
| 229327_s_at | --- | 1.17 |  |
| 205863_at | S100A12 | 1.17 |  |
| 220785_at | UTS2 | 1.17 |  |
| 207496_at | MS4A2 | 1.17 |  |
| 224496_s_at | TMEM107 | 1.17 |  |
| 228585_at | ENTPD1 | 1.17 |  |
| 217824_at | UBE2J1 | 1.17 |  |
| 210889_s_at | FCGR2B | 1.17 |  |
| 224707_at | C5orf32 | 1.17 |  |
| 1552772_at | CLEC4D | 1.17 |  |
| 204232_at | FCER1G | 1.17 |  |
| 225897_at | MARCKS | 1.17 |  |
| 33646_g_at | GM2A | 1.16 |  |
| 1555687_a_at | CLEC4C | 1.16 |  |
| 1569474_at | --- | 1.16 |  |
| 232365_at | SIAH1 | 1.16 |  |
| 227893_at | C9orf130 | 1.16 |  |
| 235564_at | ZNF117 | 1.16 |  |
| 206004_at | TGM3 | 1.16 |  |
| 200986_at | SERPING1 | 1.16 |  |
| 230323_s_at | TMEM45B | 1.16 |  |
| 228170_at | OLIG1 | 1.16 |  |
| 240103_at | --- | 1.16 |  |
| 227038_at | SGMS2 | 1.16 |  |
| 202888_s_at | ANPEP | 1.16 |  |
| 1555214_a_at | CLEC7A | 1.16 |  |
| 1556423_at | VASH1 | 1.16 |  |
| 238743_at | --- | 1.16 |  |
| 205632_s_at | PIP5K1B | 1.16 |  |
| 217826_s_at | UBE2J1 | 1.16 |  |
| 1555213_a_at | CLEC7A | 1.16 |  |
| 1557924_s_at | ALPL | 1.16 |  |
| 237459_at | --- | 1.16 |  |
| 204501_at | NOV | 1.16 |  |
| 217507_at | SLC11A1 | 1.16 |  |
| 226665_at | AHSA2 | 1.16 |  |
| 220000_at | SIGLEC5 | 1.16 |  |
| 1557543_at | --- | 1.16 |  |
| 1556896_at | LOC284751 | 1.16 |  |
| 207384_at | PGLYRP1 | 1.16 |  |
| 220467_at | --- | 1.16 |  |
| 239258_at | --- | 1.16 |  |
| 217825_s_at | UBE2J1 | 1.16 |  |
| 204861_s_at | NAIP | 1.16 |  |
| 205040_at | ORM1 | 1.16 |  |
| 1569408_at | EIF2C4 | 1.16 |  |
| 202086_at | MX1 | 1.16 |  |
| 206574_s_at | PTP4A3 | 1.16 |  |
| 243683_at | MORF4L2 | 1.16 |  |
| 202976_s_at | RHOBTB3 | 1.16 |  |
| 1560797_s_at | --- | 1.15 |  |
| 1552807_a_at | SIGLEC10 /// SIGLEC12 | 1.15 |  |
| 208304_at | CCR3 | 1.15 |  |
| 227883_at | FLJ36031 | 1.15 |  |
| 222139_at | KIAA1466 | 1.15 |  |
| 209569_x_at | D4S234E | 1.15 |  |
| 219885_at | SLFN12 | 1.15 |  |
| 220302_at | MAK | 1.15 |  |
| 226006_at | LOC100131801 | 1.15 |  |
| 235703_at | PLB1 | 1.15 |  |
| 226650_at | ZFAND2A | 1.15 |  |
| 229635_at | --- | 1.15 |  |
| 212768_s_at | OLFM4 | 1.15 |  |
| 1553177_at | SH2D1B | 1.15 |  |
| 205114_s_at | CCL3 /// CCL3L1 /// CCL3L3 | 1.15 |  |
| 213002_at | MARCKS | 1.15 |  |
| 205594_at | ZNF652 | 1.15 |  |
| 220983_s_at | SPRY4 | 1.15 |  |
|  |  |  |  |
| **Downregulated** | |  |  |
|  |  |  |  |
| 239806_at | --- | 0.87 |  |
| 203608_at | ALDH5A1 | 0.87 |  |
| 213998_s_at | DDX17 | 0.87 |  |
| 219737_s_at | PCDH9 | 0.87 |  |
| 207651_at | GPR171 | 0.87 |  |
| 235434_at | --- | 0.87 |  |
| 202971_s_at | DYRK2 | 0.87 |  |
| 210116_at | SH2D1A | 0.87 |  |
| 217643_x_at | --- | 0.87 |  |
| 212706_at | LOC100286937 /// LOC100287164 /// RASA4 | 0.87 |  |
| 235652_at | --- | 0.87 |  |
| 204749_at | NAP1L3 | 0.87 |  |
| 205608_s_at | ANGPT1 | 0.87 |  |
| 201341_at | ENC1 | 0.87 |  |
| 204890_s_at | LCK | 0.87 |  |
| 210288_at | KLRG1 | 0.87 |  |
| 210087_s_at | MPZL1 | 0.87 |  |
| 214012_at | ERAP1 | 0.87 |  |
| 219093_at | PID1 | 0.87 |  |
| 228708_at | RAB27B | 0.87 |  |
| 221648_s_at | --- | 0.87 |  |
| 234621_at | --- | 0.87 |  |
| AFFX-HUMRGE/M10098_5_at | --- | 0.87 |  |
| 238692_at | BTBD11 | 0.87 |  |
| 223645_s_at | CYorf15B | 0.87 |  |
| 1558972_s_at | THEMIS | 0.87 |  |
| 203678_at | MTMR15 | 0.87 |  |
| 202742_s_at | PRKACB | 0.87 |  |
| 200610_s_at | NCL | 0.87 |  |
| 231152_at | INO80D | 0.87 |  |
| 235085_at | PRAGMIN | 0.87 |  |
| 214131_at | CYorf15B | 0.86 |  |
| 203819_s_at | IGF2BP3 | 0.86 |  |
| 214146_s_at | PPBP | 0.86 |  |
| 218793_s_at | SCML1 | 0.86 |  |
| 220059_at | STAP1 | 0.86 |  |
| 221564_at | PRMT2 | 0.86 |  |
| 234640_x_at | --- | 0.86 |  |
| 204567_s_at | ABCG1 | 0.86 |  |
| 205049_s_at | CD79A | 0.86 |  |
| 239287_at | --- | 0.86 |  |
| 220370_s_at | USP36 | 0.86 |  |
| 207815_at | PF4V1 | 0.86 |  |
| 212730_at | SYNM | 0.86 |  |
| 206918_s_at | CPNE1 | 0.86 |  |
| 232803_at | FLJ31958 | 0.86 |  |
| 234975_at | GSPT1 | 0.86 |  |
| 218430_s_at | RFX7 | 0.86 |  |
| 37145_at | GNLY | 0.86 |  |
| 231828_at | LOC253039 | 0.86 |  |
| 206337_at | CCR7 | 0.86 |  |
| 229778_at | C12orf39 | 0.86 |  |
| 244663_at | --- | 0.86 |  |
| 222317_at | PDE3B | 0.86 |  |
| 211149_at | UTY | 0.86 |  |
| 214551_s_at | CD7 | 0.86 |  |
| 226003_at | KIF21A | 0.86 |  |
| 209374_s_at | IGHM | 0.86 |  |
| 228195_at | C2orf88 | 0.86 |  |
| 228960_at | NARG2 | 0.86 |  |
| 228487_s_at | --- | 0.86 |  |
| 229007_at | LOC283788 | 0.86 |  |
| 230913_at | --- | 0.86 |  |
| 239726_at | ANK3 | 0.86 |  |
| 224058_s_at | HSD17B7P2 | 0.86 |  |
| 1556054_at | --- | 0.86 |  |
| 224048_at | USP44 | 0.86 |  |
| 230464_at | S1PR5 | 0.86 |  |
| 1556173_a_at | --- | 0.86 |  |
| 210370_s_at | LY9 | 0.86 |  |
| 209307_at | SWAP70 | 0.86 |  |
| 220940_at | ANKRD36B | 0.86 |  |
| 235885_at | P2RY12 | 0.86 |  |
| 241803_s_at | RP11-160N1.10 | 0.86 |  |
| 243780_at | --- | 0.86 |  |
| 203563_at | AFAP1 | 0.86 |  |
| 204581_at | CD22 | 0.86 |  |
| 241871_at | CAMK4 | 0.86 |  |
| 201631_s_at | IER3 | 0.86 |  |
| 229187_at | LOC283788 | 0.86 |  |
| 211893_x_at | CD6 | 0.86 |  |
| 232489_at | CCDC76 | 0.86 |  |
| 229513_at | STRBP | 0.86 |  |
| 244075_at | --- | 0.86 |  |
| 203196_at | ABCC4 | 0.86 |  |
| 1553856_s_at | P2RY10 | 0.86 |  |
| 212671_s_at | HLA-DQA1 /// HLA-DQA2 /// LOC100294224 /// LOC100294317 | 0.86 |  |
| 220094_s_at | CCDC90A | 0.86 |  |
| 223717_s_at | ACRBP | 0.86 |  |
| 203178_at | GATM | 0.85 |  |
| 215470_at | GTF2H2B | 0.85 |  |
| 240246_at | --- | 0.85 |  |
| 236000_s_at | --- | 0.85 |  |
| 205495_s_at | GNLY | 0.85 |  |
| 230877_at | IGHD | 0.85 |  |
| 233884_at | HIVEP3 | 0.85 |  |
| 231013_at | --- | 0.85 |  |
| 202950_at | CRYZ | 0.85 |  |
| 214615_at | P2RY10 | 0.85 |  |
| 236280_at | --- | 0.85 |  |
| 215390_at | --- | 0.85 |  |
| 221234_s_at | BACH2 | 0.85 |  |
| 241365_at | --- | 0.85 |  |
| 239208_s_at | C21orf57 | 0.85 |  |
| 235982_at | FCRL1 | 0.85 |  |
| 235400_at | FCRLA | 0.85 |  |
| 219667_s_at | BANK1 | 0.85 |  |
| 222915_s_at | BANK1 | 0.85 |  |
| 225496_s_at | SYTL2 | 0.85 |  |
| 235401_s_at | FCRLA | 0.85 |  |
| 219368_at | NAP1L2 | 0.85 |  |
| 227198_at | AFF3 | 0.85 |  |
| 1567080_s_at | CLN6 | 0.85 |  |
| 234849_at | TRA@ | 0.85 |  |
| 208602_x_at | CD6 | 0.85 |  |
| 210279_at | GPR18 | 0.85 |  |
| 205590_at | RASGRP1 | 0.85 |  |
| 228725_x_at | PRMT2 | 0.85 |  |
| 218170_at | ISOC1 | 0.85 |  |
| 201874_at | MPZL1 | 0.85 |  |
| 206283_s_at | TAL1 | 0.85 |  |
| 206928_at | ZNF124 | 0.85 |  |
| 227250_at | KREMEN1 | 0.85 |  |
| 235987_at | PRKXP1 | 0.85 |  |
| 217207_s_at | BTNL3 | 0.85 |  |
| 218935_at | EHD3 | 0.85 |  |
| 225835_at | SLC12A2 | 0.85 |  |
| 206624_at | USP9Y | 0.85 |  |
| 239043_at | ZNF404 | 0.85 |  |
| 218161_s_at | CLN6 | 0.85 |  |
| 221969_at | --- | 0.85 |  |
| 206974_at | CXCR6 | 0.85 |  |
| 217627_at | ZNF573 | 0.85 |  |
| 1553132_a_at | TC2N | 0.84 |  |
| 238649_at | PITPNC1 | 0.84 |  |
| 1553380_at | PARP15 | 0.84 |  |
| 201875_s_at | MPZL1 | 0.84 |  |
| 215314_at | --- | 0.84 |  |
| 204731_at | TGFBR3 | 0.84 |  |
| 204794_at | DUSP2 | 0.84 |  |
| 230245_s_at | LOC283663 | 0.84 |  |
| 235104_at | ERAP2 | 0.84 |  |
| 228390_at | --- | 0.84 |  |
| 215652_at | SDHD | 0.84 |  |
| 201108_s_at | THBS1 | 0.84 |  |
| 228498_at | --- | 0.84 |  |
| 231647_s_at | FCRL5 | 0.84 |  |
| 237875_at | --- | 0.84 |  |
| 1564435_a_at | KRT72 | 0.84 |  |
| 235157_at | --- | 0.84 |  |
| 210432_s_at | SCN3A | 0.84 |  |
| 224823_at | MYLK | 0.84 |  |
| 205001_s_at | DDX3Y | 0.84 |  |
| 210461_s_at | ABLIM1 | 0.84 |  |
| 212958_x_at | PAM | 0.84 |  |
| 201109_s_at | THBS1 | 0.84 |  |
| 225046_at | LOC389831 | 0.84 |  |
| 238458_at | EFHA2 | 0.84 |  |
| 228643_at | --- | 0.84 |  |
| 224009_x_at | DHRS9 | 0.84 |  |
| 226625_at | TGFBR3 | 0.84 |  |
| 222347_at | LOC644450 | 0.84 |  |
| 204081_at | NRGN | 0.84 |  |
| 215262_at | --- | 0.84 |  |
| 212805_at | PRUNE2 | 0.84 |  |
| 227462_at | ERAP2 | 0.84 |  |
| 1568658_at | C2orf74 | 0.84 |  |
| 208456_s_at | RRAS2 | 0.84 |  |
| 244482_at | --- | 0.84 |  |
| 212827_at | IGHM | 0.84 |  |
| 242052_at | --- | 0.84 |  |
| 226223_at | --- | 0.83 |  |
| 223952_x_at | DHRS9 | 0.83 |  |
| 211600_at | PTPRO | 0.83 |  |
| 217418_x_at | MS4A1 | 0.83 |  |
| 1552677_a_at | DIP2A | 0.83 |  |
| 220486_x_at | TMEM164 | 0.83 |  |
| AFFX-HUMRGE/M10098_M_at | --- | 0.83 |  |
| 1556172_at | --- | 0.83 |  |
| 200629_at | WARS | 0.83 |  |
| 223202_s_at | TMEM164 | 0.83 |  |
| 201059_at | CTTN | 0.83 |  |
| 1553196_a_at | FCRL3 | 0.83 |  |
| 210356_x_at | MS4A1 | 0.83 |  |
| 231093_at | FCRL3 | 0.83 |  |
| 206700_s_at | KDM5D | 0.83 |  |
| 234151_at | --- | 0.83 |  |
| 228722_at | PRMT2 | 0.83 |  |
| 206390_x_at | PF4 | 0.83 |  |
| 206655_s_at | GP1BB /// SEPT5 | 0.83 |  |
| 230760_at | ZFY | 0.83 |  |
| 208792_s_at | CLU | 0.83 |  |
| 228592_at | MS4A1 | 0.83 |  |
| 200628_s_at | WARS | 0.83 |  |
| AFFX-HUMRGE/M10098_3_at | --- | 0.83 |  |
| 224310_s_at | BCL11B | 0.83 |  |
| 1554273_a_at | ERAP2 | 0.83 |  |
| 204160_s_at | ENPP4 | 0.82 |  |
| 215111_s_at | TSC22D1 | 0.82 |  |
| 232914_s_at | SYTL2 | 0.82 |  |
| 218711_s_at | SDPR | 0.82 |  |
| 215101_s_at | CXCL5 | 0.82 |  |
| 213674_x_at | IGHD | 0.82 |  |
| 1558882_at | LOC401233 | 0.82 |  |
| 217422_s_at | CD22 | 0.82 |  |
| 1564139_at | LOC144571 | 0.82 |  |
| 1557733_a_at | --- | 0.82 |  |
| 242565_x_at | C21orf57 | 0.82 |  |
| 208791_at | CLU | 0.82 |  |
| 237839_at | --- | 0.82 |  |
| 223201_s_at | TMEM164 | 0.82 |  |
| 232028_at | ZNF678 | 0.82 |  |
| 201340_s_at | ENC1 | 0.82 |  |
| 210206_s_at | DDX11 | 0.82 |  |
| 219759_at | ERAP2 | 0.82 |  |
| 241420_at | --- | 0.82 |  |
| 242457_at | --- | 0.81 |  |
| 242476_at | --- | 0.81 |  |
| 1563357_at | --- | 0.81 |  |
| 214971_s_at | ST6GAL1 | 0.81 |  |
| 202336_s_at | PAM | 0.81 |  |
| 206494_s_at | ITGA2B | 0.81 |  |
| 1559252_a_at | C20orf29 | 0.81 |  |
| 211064_at | ZNF493 | 0.81 |  |
| 214414_x_at | HBA1 /// HBA2 | 0.81 |  |
| 216069_at | --- | 0.81 |  |
| 1554636_at | --- | 0.81 |  |
| 209841_s_at | LRRN3 | 0.81 |  |
| 204410_at | EIF1AY | 0.81 |  |
| 231979_at | --- | 0.81 |  |
| 228599_at | MS4A1 | 0.81 |  |
| 233011_at | ANXA1 | 0.81 |  |
| 229614_at | ZNF320 | 0.81 |  |
| 206785_s_at | KLRC1 /// KLRC2 | 0.81 |  |
| 216191_s_at | TRA@ /// TRD@ | 0.81 |  |
| 217143_s_at | TRA@ /// TRD@ | 0.81 |  |
| 222196_at | LOC286434 | 0.81 |  |
| 227559_at | NDUFAF4 | 0.81 |  |
| 200665_s_at | SPARC | 0.81 |  |
| 205767_at | EREG | 0.80 |  |
| 228492_at | USP9Y | 0.80 |  |
| 231798_at | NOG | 0.80 |  |
| 204627_s_at | ITGB3 | 0.80 |  |
| 209840_s_at | LRRN3 | 0.80 |  |
| 204838_s_at | MLH3 | 0.80 |  |
| 218345_at | TMEM176A | 0.80 |  |
| 244631_at | LOC389834 | 0.80 |  |
| 234632_x_at | --- | 0.80 |  |
| 203414_at | MMD | 0.79 |  |
| 243450_at | --- | 0.79 |  |
| 1553608_a_at | NCRNA00189 | 0.79 |  |
| 229704_at | --- | 0.79 |  |
| 207072_at | IL18RAP | 0.79 |  |
| 225767_at | --- | 0.79 |  |
| 227199_at | DIP2A | 0.79 |  |
| 215555_at | --- | 0.79 |  |
| 217712_at | LOC389906 | 0.79 |  |
| 211144_x_at | TARP /// TRGC2 | 0.78 |  |
| 222717_at | SDPR | 0.78 |  |
| 215806_x_at | TARP /// TRGC2 | 0.78 |  |
| 210732_s_at | LGALS8 | 0.78 |  |
| 242577_at | LOC389834 | 0.78 |  |
| 205382_s_at | CFD | 0.78 |  |
| 216920_s_at | TARP /// TRGC2 | 0.78 |  |
| 205609_at | ANGPT1 | 0.77 |  |
| 202286_s_at | TACSTD2 | 0.77 |  |
| 209813_x_at | TARP | 0.77 |  |
| 221211_s_at | C21orf7 | 0.77 |  |
| 219825_at | CYP26B1 | 0.77 |  |
| 201909_at | RPS4Y1 | 0.77 |  |
| 204018_x_at | HBA1 /// HBA2 | 0.77 |  |
| 235172_at | --- | 0.77 |  |
| 209458_x_at | HBA1 /// HBA2 | 0.76 |  |
| 203680_at | PRKAR2B | 0.76 |  |
| 1568983_a_at | --- | 0.76 |  |
| 235535_x_at | FRG1 /// FRG1B /// LOC642236 | 0.76 |  |
| 211696_x_at | HBB | 0.76 |  |
| 211745_x_at | HBA1 /// HBA2 | 0.76 |  |
| 230690_at | TUBB1 | 0.76 |  |
| 225283_at | ARRDC4 | 0.75 |  |
| 217414_x_at | HBA1 /// HBA2 | 0.75 |  |
| 235274_at | --- | 0.75 |  |
| 206385_s_at | ANK3 | 0.74 |  |
| 201110_s_at | THBS1 | 0.74 |  |
| 217232_x_at | HBB | 0.74 |  |
| 209301_at | CA2 | 0.74 |  |
| 208601_s_at | TUBB1 | 0.73 |  |
| 204115_at | GNG11 | 0.73 |  |
| 243495_s_at | --- | 0.73 |  |
| 226558_at | LOC389834 | 0.73 |  |
| 215666_at | HLA-DRB4 | 0.73 |  |
| 211699_x_at | HBA1 /// HBA2 | 0.72 |  |
| 1569955_at | --- | 0.72 |  |
| 205000_at | DDX3Y | 0.72 |  |
| 1559156_at | --- | 0.71 |  |
| 229026_at | --- | 0.71 |  |
| 209116_x_at | HBB | 0.71 |  |
| 213005_s_at | KANK1 | 0.70 |  |
| 204409_s_at | EIF1AY | 0.70 |  |
| 201694_s_at | EGR1 | 0.70 |  |
| 214974_x_at | CXCL5 | 0.69 |  |
| 208151_x_at | DDX17 | 0.68 |  |
| 209706_at | NKX3-1 | 0.67 |  |
| 208719_s_at | DDX17 | 0.67 |  |
| 209686_at | S100B | 0.65 |  |
| 226475_at | FAM118A | 0.64 |  |
| 209728_at | HLA-DRB4 | 0.60 |  |
| 220532_s_at | TMEM176B | 0.57 |  |
| 227404_s_at | EGR1 | 0.57 |  |
| 219629_at | FAM118A | 0.53 |  |
|  |  |  |  |
| *** Ratio: MDD/HC** | |  |  |
