# Supplementary material for: Alterations in leukocyte transcriptional control pathway activity associated with major depressive disorder and antidepressant treatment
Source: Transl Psychiatry. 2016 May 24;6(5):e821–. doi: 10.1038/tp.2016.79 (PMC5070063; doi:10.1038/tp.2016.79)
Supplement: Supplementary Table 3 [file tp201679x3.docx]

**Supporting Information Table S3: Transcripts differentially expressed in MDD subjects after 8 weeks of antidepressant treatment vs baseline**

| **Probe Set ID** | **Gene Symbol** | **Fold-difference*** | |
| --- | --- | --- | --- |
| 233011_at | ANXA1 | 1.54 |  |
| 205000_at | DDX3Y | 1.48 |  |
| 205048_s_at | PSPH | 1.47 |  |
| 204439_at | IFI44L | 1.43 |  |
| 226702_at | CMPK2 | 1.41 |  |
| 213797_at | RSAD2 | 1.38 |  |
| 204409_s_at | EIF1AY | 1.38 |  |
| 205552_s_at | OAS1 | 1.37 |  |
| 201909_at | RPS4Y1 | 1.37 |  |
| 202869_at | OAS1 | 1.35 |  |
| 225767_at | --- | 1.35 |  |
| 242625_at | RSAD2 | 1.35 |  |
| 202411_at | IFI27 | 1.34 |  |
| 203290_at | HLA-DQA1 | 1.34 |  |
| 218400_at | OAS3 | 1.34 |  |
| 224225_s_at | ETV7 | 1.34 |  |
| 208450_at | LGALS2 | 1.32 |  |
| 206881_s_at | LILRA3 | 1.31 |  |
| 204533_at | CXCL10 | 1.31 |  |
| 236156_at | LIPA | 1.31 |  |
| 217933_s_at | LAP3 | 1.29 |  |
| 227347_x_at | HES4 | 1.29 |  |
| 226103_at | NEXN | 1.29 |  |
| 1554273_a_at | ERAP2 | 1.28 |  |
| 204451_at | FZD1 | 1.28 |  |
| 211776_s_at | EPB41L3 | 1.28 |  |
| 219607_s_at | MS4A4A | 1.28 |  |
| 225762_x_at | LOC284801 | 1.28 |  |
| 214453_s_at | IFI44 | 1.27 |  |
| 211864_s_at | MYOF | 1.27 |  |
| 219519_s_at | SIGLEC1 | 1.27 |  |
| 205826_at | MYOM2 | 1.27 |  |
| 226558_at | LOC389834 | 1.27 |  |
| 202007_at | NID1 | 1.27 |  |
| 206624_at | USP9Y | 1.27 |  |
| 239979_at | --- | 1.26 |  |
| 202086_at | MX1 | 1.26 |  |
| 238439_at | ANKRD22 | 1.26 |  |
| 226517_at | BCAT1 | 1.26 |  |
| 228492_at | USP9Y | 1.26 |  |
| 212845_at | SAMD4A | 1.26 |  |
| 210873_x_at | APOBEC3A | 1.26 |  |
| 203153_at | IFIT1 | 1.26 |  |
| 209684_at | RIN2 | 1.25 |  |
| 235885_at | P2RY12 | 1.25 |  |
| 205483_s_at | ISG15 | 1.25 |  |
| 220146_at | TLR7 | 1.25 |  |
| 214059_at | IFI44 | 1.25 |  |
| 218559_s_at | MAFB | 1.25 |  |
| 229391_s_at | FAM26F | 1.24 |  |
| 1553962_s_at | RHOB | 1.24 |  |
| 212681_at | EPB41L3 | 1.24 |  |
| 206700_s_at | KDM5D | 1.24 |  |
| 206710_s_at | EPB41L3 | 1.24 |  |
| 1552309_a_at | NEXN | 1.24 |  |
| 219872_at | C4orf18 | 1.23 |  |
| 219759_at | ERAP2 | 1.23 |  |
| 205660_at | OASL | 1.23 |  |
| 227609_at | EPSTI1 | 1.23 |  |
| 204415_at | IFI6 | 1.23 |  |
| 202145_at | LY6E | 1.23 |  |
| 208436_s_at | IRF7 | 1.23 |  |
| 206133_at | XAF1 | 1.23 |  |
| 231578_at | GBP1 | 1.23 |  |
| 242427_at | WAC | 1.23 |  |
| 204972_at | OAS2 | 1.22 |  |
| 224973_at | FAM46A | 1.22 |  |
| 1560800_at | --- | 1.22 |  |
| 226184_at | FMNL2 | 1.22 |  |
| 204858_s_at | TYMP | 1.22 |  |
| 213348_at | CDKN1C | 1.22 |  |
| 225285_at | BCAT1 | 1.22 |  |
| 235385_at | --- | 1.22 |  |
| 230645_at | FRMD3 | 1.22 |  |
| 44673_at | SIGLEC1 | 1.22 |  |
| 219863_at | HERC5 | 1.22 |  |
| 210435_at | ARL17 | 1.22 |  |
| 201798_s_at | MYOF | 1.22 |  |
| 231769_at | FBXO6 | 1.21 |  |
| 213182_x_at | CDKN1C | 1.21 |  |
| 235643_at | SAMD9L | 1.21 |  |
| 240646_at | GIMAP8 | 1.21 |  |
| 220492_s_at | OTOF | 1.21 |  |
| 215891_s_at | GM2A | 1.21 |  |
| 222670_s_at | MAFB | 1.21 |  |
| 219534_x_at | CDKN1C | 1.21 |  |
| 206553_at | OAS2 | 1.21 |  |
| 219574_at | 40603 | 1.21 |  |
| 242234_at | XAF1 | 1.21 |  |
| 219629_at | FAM118A | 1.21 |  |
| 201015_s_at | JUP | 1.2 |  |
| 235104_at | ERAP2 | 1.2 |  |
| 210935_s_at | WDR1 | 1.2 |  |
| 223599_at | TRIM6 | 1.2 |  |
| 209616_s_at | CES1 | 1.2 |  |
| 209555_s_at | CD36 | 1.2 |  |
| 205382_s_at | CFD | 1.2 |  |
| 210797_s_at | OASL | 1.2 |  |
| 240551_at | ZBTB45 | 1.2 |  |
| 233917_s_at | MOV10 | 1.2 |  |
| 242288_s_at | EMILIN2 | 1.2 |  |
| 1555728_a_at | MS4A4A | 1.2 |  |
| 204560_at | FKBP5 | 1.2 |  |
| 222760_at | ZNF703 | 1.2 |  |
| 219211_at | USP18 | 1.2 |  |
| 214452_at | BCAT1 | 1.19 |  |
| 210362_x_at | PML | 1.19 |  |
| 200986_at | SERPING1 | 1.19 |  |
| 235276_at | EPSTI1 | 1.19 |  |
| 228439_at | BATF2 | 1.19 |  |
| 207091_at | P2RX7 | 1.19 |  |
| 227792_at | ITPRIPL2 | 1.19 |  |
| 234621_at | --- | 1.19 |  |
| 203771_s_at | BLVRA | 1.19 |  |
| 212224_at | ALDH1A1 | 1.19 |  |
| 203236_s_at | LGALS9 | 1.19 |  |
| 214038_at | CCL8 | 1.19 |  |
| 229390_at | FAM26F | 1.19 |  |
| 230314_at | --- | 1.19 |  |
| 229937_x_at | LILRB1 | 1.19 |  |
| 201646_at | SCARB2 | 1.19 |  |
| 204747_at | IFIT3 | 1.19 |  |
| 209213_at | CBR1 | 1.19 |  |
| 227462_at | ERAP2 | 1.19 |  |
| 243348_at | --- | 1.19 |  |
| 228617_at | XAF1 | 1.19 |  |
| 218986_s_at | DDX60 | 1.19 |  |
| 219684_at | RTP4 | 1.19 |  |
| 228697_at | HINT3 | 1.19 |  |
| 1567628_at | CD74 | 1.18 |  |
| 232383_at | TFEC | 1.18 |  |
| 239591_at | LRRC37A3 | 1.18 |  |
| 204224_s_at | GCH1 | 1.18 |  |
| 219209_at | IFIH1 | 1.18 |  |
| 206715_at | TFEC | 1.18 |  |
| 219570_at | KIF16B | 1.18 |  |
| 228937_at | C13orf31 | 1.18 |  |
| 219895_at | FAM70A | 1.18 |  |
| 1558214_s_at | CTNNA1 | 1.18 |  |
| 236995_x_at | TFEC | 1.18 |  |
| 241347_at | KIAA1618 | 1.18 |  |
| 229067_at | SRGAP2P1 | 1.18 |  |
| 230180_at | --- | 1.18 |  |
| 229510_at | MS4A14 | 1.18 |  |
| 216894_x_at | CDKN1C | 1.18 |  |
| 204410_at | EIF1AY | 1.18 |  |
| 200935_at | CALR | 1.18 |  |
| 230741_at | --- | 1.18 |  |
| 212671_s_at | HLA-DQA1 /// HLA-DQA2 /// LOC100294224 /// LOC100294317 | 1.18 |  |
| 33646_g_at | GM2A | 1.18 |  |
| 205114_s_at | CCL3 /// CCL3L1 /// CCL3L3 | 1.18 |  |
| 202269_x_at | GBP1 | 1.18 |  |
| 222816_s_at | ZCCHC2 | 1.17 |  |
| 224983_at | SCARB2 | 1.17 |  |
| 56256_at | SIDT2 | 1.17 |  |
| 209417_s_at | IFI35 | 1.17 |  |
| 209499_x_at | TNFSF12-TNFSF13 /// TNFSF13 | 1.17 |  |
| 221288_at | GPR22 | 1.17 |  |
| 1568619_s_at | ITPRIPL2 | 1.17 |  |
| 223849_s_at | MOV10 | 1.17 |  |
| 222509_s_at | ZNF672 | 1.17 |  |
| 208146_s_at | CPVL | 1.17 |  |
| 200628_s_at | WARS | 1.17 |  |
| 205569_at | LAMP3 | 1.17 |  |
| 214770_at | MSR1 | 1.17 |  |
| 202434_s_at | CYP1B1 | 1.17 |  |
| AFFX-HUMISGF3A/M97935_MA_at | STAT1 | 1.17 |  |
| 227798_at | SMAD1 | 1.17 |  |
| 235400_at | FCRLA | 1.17 |  |
| 223645_s_at | CYorf15B | 1.17 |  |
| 204392_at | CAMK1 | 1.17 |  |
| 226668_at | WDSUB1 | 1.17 |  |
| 204039_at | CEBPA | 1.17 |  |
| 205936_s_at | HK3 | 1.17 |  |
| 222154_s_at | SPATS2L | 1.17 |  |
| 239412_at | IRF5 | 1.17 |  |
| 219288_at | C3orf14 | 1.17 |  |
| 226603_at | SAMD9L | 1.17 |  |
| 206214_at | PLA2G7 | 1.17 |  |
| 215495_s_at | SAMD4A | 1.17 |  |
| 1557236_at | APOL6 | 1.17 |  |
| 215120_s_at | SAMD4A | 1.17 |  |
| 211284_s_at | GRN | 1.17 |  |
| 1555745_a_at | LYZ | 1.17 |  |
| 229115_at | DYNC1H1 | 1.17 |  |
| 229543_at | FAM26F | 1.17 |  |
| 202671_s_at | PDXK | 1.17 |  |
| 226022_at | SASH1 | 1.16 |  |
| 201647_s_at | SCARB2 | 1.16 |  |
| AFFX-HUMISGF3A/M97935_5_at | STAT1 | 1.16 |  |
| 211495_x_at | TNFSF12-TNFSF13 /// TNFSF13 | 1.16 |  |
| 1556423_at | VASH1 | 1.16 |  |
| 229450_at | IFIT3 | 1.16 |  |
| 226475_at | FAM118A | 1.16 |  |
| 211138_s_at | KMO | 1.16 |  |
| 64408_s_at | CALML4 | 1.16 |  |
| 202430_s_at | PLSCR1 | 1.16 |  |
| 230550_at | MS4A6A | 1.16 |  |
| 203922_s_at | CYBB | 1.16 |  |
| 243271_at | --- | 1.16 |  |
| 224840_at | FKBP5 | 1.16 |  |
| 221679_s_at | ABHD6 | 1.16 |  |
| 202943_s_at | NAGA | 1.16 |  |
| 218232_at | C1QA | 1.16 |  |
| 206928_at | ZNF124 | 1.16 |  |
| 210705_s_at | TRIM5 | 1.16 |  |
| 228343_at | POU2F2 | 1.16 |  |
| 217502_at | IFIT2 | 1.16 |  |
| 205306_x_at | KMO | 1.16 |  |
| 230036_at | SAMD9L | 1.16 |  |
| 219716_at | APOL6 | 1.16 |  |
| 223344_s_at | MS4A7 | 1.16 |  |
| 216950_s_at | FCGR1A /// FCGR1C | 1.16 |  |
| 202787_s_at | MAPKAPK3 | 1.16 |  |
| 200766_at | CTSD | 1.16 |  |
| 210895_s_at | CD86 | 1.16 |  |
| AFFX-HUMISGF3A/M97935_MB_at | STAT1 | 1.16 |  |
| 215049_x_at | CD163 | 1.16 |  |
| 201641_at | BST2 | 1.16 |  |
| 208890_s_at | PLXNB2 | 1.16 |  |
| 214511_x_at | FCGR1B | 1.16 |  |
| 224856_at | FKBP5 | 1.16 |  |
| 224924_at | TTC7A | 1.16 |  |
| 229383_at | --- | 1.16 |  |
| 225291_at | PNPT1 | 1.16 |  |
| 226069_at | PRICKLE1 | 1.16 |  |
| 201761_at | MTHFD2 | 1.16 |  |
| 211729_x_at | BLVRA | 1.15 |  |
| 206491_s_at | NAPA | 1.15 |  |
| 205170_at | STAT2 | 1.15 |  |
| 203595_s_at | IFIT5 | 1.15 |  |
| 205654_at | C4BPA | 1.15 |  |
| 228176_at | S1PR3 | 1.15 |  |
| 200629_at | WARS | 1.15 |  |
| 223991_s_at | GALNT2 /// LOC100132910 | 1.15 |  |
| 228624_at | TMEM144 | 1.15 |  |
| 223204_at | C4orf18 | 1.15 |  |
| 244398_x_at | ZNF684 | 1.15 |  |
| 210314_x_at | TNFSF13 | 1.15 |  |
| 225651_at | UBE2E2 | 1.15 |  |
| 225869_s_at | UNC93B1 | 1.15 |  |
| 231577_s_at | GBP1 | 1.15 |  |
| 204276_at | TK2 | 1.15 |  |
| 203923_s_at | CYBB | 1.15 |  |
| 213294_at | --- | 1.15 |  |
| 223343_at | MS4A7 | 1.15 |  |
| 229893_at | FRMD3 | 1.15 |  |
| 238327_at | ODF3B | 1.15 |  |
| 225188_at | RAPH1 | 1.15 |  |
| 1552486_s_at | LACTB | 1.15 |  |
| 205237_at | FCN1 | 1.15 |  |
|  |  |  |  |
| **Downregulated** | |  |  |
|  |  |  |  |
| 205594_at | ZNF652 | 0.87 |  |
| 1552622_s_at | LOC441259 /// POLR2J2 | 0.87 |  |
| 214321_at | NOV | 0.87 |  |
| 228248_at | RICTOR | 0.87 |  |
| 1559190_s_at | RDH13 | 0.87 |  |
| 1554690_a_at | TACC1 | 0.87 |  |
| 232862_at | ABHD5 | 0.87 |  |
| 1562481_at | --- | 0.87 |  |
| 1564568_at | --- | 0.87 |  |
| 231996_at | N4BP2 | 0.87 |  |
| 1569392_at | TECR | 0.87 |  |
| 1568815_a_at | DDX50 | 0.87 |  |
| 211696_x_at | HBB | 0.87 |  |
| 233202_at | CNTNAP3 | 0.87 |  |
| 1569503_at | HEATR5B | 0.87 |  |
| 243016_at | --- | 0.87 |  |
| 1560033_at | MCCC2 | 0.87 |  |
| 237377_at | --- | 0.87 |  |
| 220983_s_at | SPRY4 | 0.87 |  |
| 1564970_at | SETDB2 | 0.87 |  |
| 1562836_at | --- | 0.87 |  |
| 221477_s_at | SOD2 | 0.87 |  |
| 1555485_s_at | FAM153B | 0.87 |  |
| 224322_at | ARID4B | 0.87 |  |
| 244061_at | --- | 0.87 |  |
| 240336_at | HBM | 0.87 |  |
| 1569477_at | --- | 0.87 |  |
| 235094_at | --- | 0.86 |  |
| 238983_at | NSUN7 | 0.86 |  |
| 210724_at | EMR3 | 0.86 |  |
| 237943_at | --- | 0.86 |  |
| 230083_at | USP53 | 0.86 |  |
| 1554638_at | ZFYVE16 | 0.86 |  |
| 216841_s_at | SOD2 | 0.86 |  |
| 1558078_at | --- | 0.86 |  |
| 240265_at | TRAF3IP3 | 0.86 |  |
| 216050_at | --- | 0.86 |  |
| 219227_at | CCNJL | 0.86 |  |
| 231886_at | DKFZP434B2016 | 0.86 |  |
| 1556185_a_at | --- | 0.86 |  |
| 207384_at | PGLYRP1 | 0.86 |  |
| 210244_at | CAMP | 0.86 |  |
| 1562467_at | --- | 0.86 |  |
| 224098_at | --- | 0.86 |  |
| 225155_at | SNHG5 | 0.86 |  |
| 1558515_at | NCRNA00182 | 0.86 |  |
| 227223_at | LOC643167 /// RBM39 | 0.86 |  |
| 215029_at | --- | 0.86 |  |
| 200606_at | DSP | 0.86 |  |
| 236164_at | FLJ10038 | 0.86 |  |
| 220528_at | VNN3 | 0.86 |  |
| 220421_at | BTNL8 | 0.86 |  |
| 215694_at | SPATA5L1 | 0.86 |  |
| 238320_at | NEAT1 | 0.86 |  |
| 220712_at | C8orf60 | 0.86 |  |
| 233724_at | ARNT | 0.86 |  |
| 227722_at | RPS23 | 0.86 |  |
| 1554703_at | ARHGEF10 | 0.86 |  |
| 233302_at | --- | 0.86 |  |
| 217232_x_at | HBB | 0.86 |  |
| 240347_at | --- | 0.86 |  |
| 213549_at | --- | 0.86 |  |
| 232584_at | --- | 0.86 |  |
| 206522_at | MGAM | 0.86 |  |
| 237180_at | PSME4 | 0.86 |  |
| 219797_at | MGAT4A | 0.86 |  |
| 236495_at | --- | 0.86 |  |
| 238723_at | ATXN3 | 0.85 |  |
| 1566966_at | --- | 0.85 |  |
| 1565716_at | FUS | 0.85 |  |
| 238449_at | LOC595101 | 0.85 |  |
| 213906_at | MYBL1 | 0.85 |  |
| 202948_at | IL1R1 | 0.85 |  |
| 240156_at | --- | 0.85 |  |
| 206177_s_at | ARG1 | 0.85 |  |
| 1557924_s_at | ALPL | 0.85 |  |
| 41469_at | PI3 | 0.85 |  |
| 242191_at | NBPF10 | 0.85 |  |
| 204419_x_at | HBG1 /// HBG2 | 0.85 |  |
| 224576_at | ERGIC1 | 0.85 |  |
| 209116_x_at | HBB | 0.85 |  |
| 204750_s_at | DSC2 | 0.85 |  |
| 1558504_at | --- | 0.85 |  |
| 215047_at | TRIM58 | 0.85 |  |
| 231108_at | FUS | 0.85 |  |
| 1557996_at | LOC100132832 /// PMS2L1 /// PMS2L2 | 0.85 |  |
| 204848_x_at | HBG1 /// HBG2 | 0.85 |  |
| 240830_at | SCARNA17 | 0.85 |  |
| 244042_x_at | --- | 0.85 |  |
| 213515_x_at | HBG1 /// HBG2 | 0.85 |  |
| 237322_at | MIAT | 0.84 |  |
| 237510_at | --- | 0.84 |  |
| 203021_at | SLPI | 0.84 |  |
| 1562307_at | --- | 0.84 |  |
| 1569955_at | --- | 0.84 |  |
| 1569512_at | --- | 0.84 |  |
| 206834_at | HBD | 0.84 |  |
| 207802_at | CRISP3 | 0.84 |  |
| 201110_s_at | THBS1 | 0.84 |  |
| 233217_at | --- | 0.84 |  |
| 213478_at | RP1-21O18.1 | 0.84 |  |
| 229010_at | CBL | 0.84 |  |
| 1570165_at | --- | 0.84 |  |
| 1568964_x_at | SPN | 0.84 |  |
| 216782_at | --- | 0.84 |  |
| 203887_s_at | THBD | 0.84 |  |
| 206676_at | CEACAM8 | 0.84 |  |
| 234044_at | --- | 0.84 |  |
| 209369_at | ANXA3 | 0.83 |  |
| 216834_at | RGS1 | 0.83 |  |
| 237459_at | --- | 0.83 |  |
| 225119_at | CHMP4B | 0.83 |  |
| 222529_at | SLC25A37 | 0.83 |  |
| 235607_at | --- | 0.83 |  |
| 215779_s_at | HIST1H2BG | 0.83 |  |
| 219179_at | DACT1 | 0.83 |  |
| 1556942_at | --- | 0.83 |  |
| 203691_at | PI3 | 0.83 |  |
| 215783_s_at | ALPL | 0.83 |  |
| 1561893_at | --- | 0.82 |  |
| 216813_at | --- | 0.82 |  |
| 227062_at | NEAT1 | 0.82 |  |
| 219730_at | MED18 | 0.82 |  |
| 1561276_at | DOCK5 | 0.82 |  |
| 226762_at | PURB | 0.82 |  |
| 231484_at | --- | 0.81 |  |
| 244065_at | CNTNAP3B | 0.81 |  |
| 1565743_at | --- | 0.81 |  |
| 222528_s_at | SLC25A37 | 0.81 |  |
| 1561128_at | --- | 0.81 |  |
| 209480_at | HLA-DQB1 | 0.81 |  |
| 202018_s_at | LTF | 0.81 |  |
| 1553542_at | CCDC125 | 0.81 |  |
| 225742_at | MDM4 | 0.8 |  |
| 205727_at | TEP1 | 0.8 |  |
| 236439_at | --- | 0.8 |  |
| 215586_at | --- | 0.8 |  |
| 209791_at | PADI2 | 0.8 |  |
| 1565976_at | --- | 0.79 |  |
| 215224_at | SNORA21 | 0.79 |  |
| 226817_at | DSC2 | 0.79 |  |
| 207329_at | MMP8 | 0.79 |  |
| 228582_x_at | --- | 0.79 |  |
| 232412_at | FBXL20 | 0.79 |  |
| 241869_at | APOL6 | 0.78 |  |
| 212531_at | LCN2 | 0.78 |  |
| 223796_at | CNTNAP3 | 0.78 |  |
| 241843_at | SNORA28 | 0.78 |  |
| 205033_s_at | DEFA1 /// DEFA1B /// DEFA3 | 0.77 |  |
| 204751_x_at | DSC2 | 0.77 |  |
| 205118_at | FPR1 | 0.77 |  |
| 207735_at | RNF125 | 0.77 |  |
| 215262_at | --- | 0.77 |  |
| 215123_at | LOC100288332 /// LOC100288583 /// NPIPL3 | 0.77 |  |
| 224589_at | XIST | 0.76 |  |
| 1562283_at | --- | 0.76 |  |
| 231688_at | MMP8 | 0.75 |  |
| 224588_at | XIST | 0.75 |  |
| 243134_at | --- | 0.75 |  |
| 221728_x_at | XIST | 0.73 |  |
| 212768_s_at | OLFM4 | 0.73 |  |
| 214218_s_at | XIST | 0.71 |  |
| 206632_s_at | APOBEC3B | 0.71 |  |
| 227671_at | XIST | 0.71 |  |
| 210387_at | HIST1H2BG | 0.7 |  |
| 205069_s_at | ARHGAP26 | 0.7 |  |
| 224559_at | MALAT1 | 0.69 |  |
| 224590_at | XIST | 0.65 |  |
| 1558048_x_at | --- | 0.48 |  |
|  |  |  |  |
| ***Ratio 8wk/baseline** | |  |  |
