# Supplementary material for: Alterations in leukocyte transcriptional control pathway activity associated with major depressive disorder and antidepressant treatment
Source: Transl Psychiatry. 2016 May 24;6(5):e821–. doi: 10.1038/tp.2016.79 (PMC5070063; doi:10.1038/tp.2016.79)
Supplement: Supplementary Table 4 [file tp201679x4.docx]

**Supporting Information Table S4: Quantitative PCR of transcripts identified as being differentially expressed in MDD vs Healthy Control Subjects**

| **GENE** | **Fold Difference**  **Healthy Control: MDD** | **P**  **Value** |
| --- | --- | --- |
| DDX17 | **0.34** | **.0001** |
| DSC2 | **1.47** | **.0005** |
| EGR1 | **0.75** | **.0001** |
| FAM118 | **0.85** | **.0121** |
| IFI44 | **0.78** | **.9871** |
| IFI44L | **1.28** | **.0239** |
| MX1 | **1.22** | **.0064** |
| PRKAR2B | **0.88** | **.0254** |
| APOBEC3B | **1.05** | **.2472** |
| MMP8 | **1.58** | **.0010** |
| XIST | **2.67** | **.0001** |
